# Cognitive Changes and Promotion in Parkinson's Disease

**DOI:** 10.1155/2018/9872183

**Published:** 2018-08-01

**Authors:** XiaoPing Wang, XiJin Wang, HaiBo Chen, Sarah Duff Canning, Xingguang Luo

**Affiliations:** ^1^Tongren Hospital, Shanghai Jiao Tong University Medical School, Shanghai 200336, China; ^2^Shanghai Jiao Tong University Medical School, Shanghai 200092, China; ^3^National Center of Gerontology, Beijing Hospital, Beijing 100730, China; ^4^University of Toronto, Toronto, ON, Canada M5W 1G3; ^5^Yale University School of Medicine, New Haven, CT 20802, USA

Parkinson's disease (PD) has been recognized as a multisystemic neurodegenerative disorder with typical motor symptoms, including static tremor, bradykinesia, rigidity, postural instability, and gait difficulty. In addition to the defining dopamine-related motor symptoms, however, increasing evidence has shown that PD patients often experience a series of nonmotor symptoms, including mood and behavior disorders, cognitive impairment, brain-gut-axis disorders, autonomic system failure, sensory symptoms, and sleep disturbances.

Cognitive impairment is one of the most devastating and common nonmotor symptoms of PD. People with PD exhibit more rapid decline in a number of cognitive domains, in particular, executive, attentional, and visuospatial domains, but also memory especially skill/implicit learning. As we all know, the underlying mechanism of motor symptoms of PD is depleted dopaminergic cells in the substantia nigra. In contrast, the pathophysiological basis of cognitive impairments in PD remains uncertain. Disrupted frontal-subcortical circuits due to dopaminergic neuron damage and wide deposition of *α*-synuclein, *β*-amyloid, and tau proteins might play a role. In K. Li et al.'s paper, they summarized rs-fMRI studies on cognitive function in PD and discuss the strong potential of rs-fMRI in this area. rs-fMRI can help reveal the pathophysiology of cognitive symptoms in PD, facilitate early identification of PD patients with cognitive impairment, distinguish PD dementia from dementia with Lewy bodies, and monitor and guide treatment for cognitive impairment in PD. In particular, ongoing and future longitudinal studies would enhance the ability of rs-fMRI in predicting PD dementia. In combination with other modalities such as positron emission tomography, rs-fMRI could give us more information on the underlying mechanism of cognitive deficits in PD.

Progressive supranuclear palsy (PSP) was first described as a progressive neurological disorder with motor, ocular, and cognitive features. Both PSP and PD are characterised by extrapyramidal syndromes, each of which can comprise symptoms of bradykinesia, rigidity, and/or postural instability. Clinically, it remains difficult to distinguish from Parkinson's disease (PD). In J. A. Foley et al.'s paper, they investigated whether the newly developed ECAS, designed to be used with people with even severe motor disability, was sensitive to the cognitive impairment seen in PD and PSP and able to distinguish between these two disorders. It is developed to be used with patients with even severe physical disability and thus may be suitable for detecting cognitive impairment in all motor disorders. Many of the subtests can be performed either orally or manually, with some measures corrected for motor speed, reducing the impact that physical disability may have performance on cognitive tests. It also allows the clinician to track cognitive impairment throughout the disease course, crucial for any longitudinal studies. ECAS is a quick, simple, and inexpensive test that can be used to support the differential diagnosis of PSP.

How to get cognitive training? There are two frequently used methods: standard or tailored. Standard cognitive training involves cognitive tasks that are not customised to the individual's cognitive deficits, whereas tailored cognitive training is deficit specific. In B. J. Lawrence et al.'s paper, they examined whether standard cognitive training, tailored cognitive training, transcranial direct current stimulation (tDCS), standard cognitive training  +  tDCS, or tailored cognitive training  +  tDCS improved cognitive function and functional outcomes in participants with PD and mild cognitive impairment (PD-MCI). And the outcomes improved for the groups that received standard or tailored cognitive training combined with tDCS. Participants with PD-MCI receiving cognitive training (standard or tailored) or tDCS demonstrated significant improvements on cognitive and functional outcomes, and combining these interventions provided greater therapeutic effects.

Another method, bilateral deep brain stimulation of subthalamic nucleus (STN-DBS), has been proven to be effective in improving motor symptoms in Parkinson's disease (PD) patients. However, psychiatric changes after surgery are controversial. So in Y. Wang et al.'s paper, they specifically analyzed apathy following bilateral STN-DBS in PD patients using a meta-analysis. They found a significant difference between the presurgery stage and the postsurgery stage scores. STN-DBS seems to relatively worsen the condition of apathy, which may result from both the surgery target (subthalamic nucleus) and the reduction of dopaminergic medication. Thus, in J. A. Foley et al.'s paper, they examined the use of standardised neuropsychological assessment for the evaluation of surgical candidates and to identify risk factors for subsequent decline in cognition and mood. They concluded that neuropsychological assessment in a sample of patients undergoing DBS for PD is suitable for the screening of candidates and can identify baseline risk factors, which requires careful consideration before and after surgery.

This issue assembles exciting, distinguished observations into the state of the art and science, as well as emerging future topics, in this important interdisciplinary field. We hope that this special issue would attract a major attention of the peers. We would like to express our appreciation heart and soul to all of the authors and reviewers.

*XiaoPing Wang* *XiJin Wang* *HaiBo Chen* *Sarah Duff Canning* *Xingguang Luo*

